# Global prostate cancer risk associated with microplastic exposure: a statistical and machine learning analysis

**DOI:** 10.3389/fpubh.2026.1857921

**Published:** 2026-06-25

**Authors:** Ashraf M. T. Elewa, Moustafa Gamal Snousy, Fatimah M. Alghamdi, Gamal A. Abd-Elmougod, Alia A. El Shahawy, Amany Fahmi Mohamed, Abdelazim Negm, Ahmed M. Saqr, Hussein M. Elshafie

**Affiliations:** 1 Geology Department, Faculty of Science, Minia University, El-Minia, Egypt; 2 Egyptian Petroleum Sector, Petrotrade Co., Cairo, Egypt; 3 Department of Mathematical Sciences, College of Science, Princess Nourah bint Abdulrahman University, Riyadh, Saudi Arabia; 4 Department of Mathematics, Faculty of Science, Islamic University of Madinah, Madinah, Saudi Arabia; 5 Department of Medical Microbiology and Immunology, Faculty of Medicine, Zagazig University, Zagazig, Egypt; 6 Computer Science Department, Faculty of Science, Qena University, Qena, Egypt; 7 Business Information System Department, Sadat Academy for Administrative Sciences, Minia, Egypt; 8 Department of Water and Water Structures Engineering, Faculty of Engineering, Zagazig University, Zagazig, Egypt; 9 Irrigation and Hydraulics Department, Faculty of Engineering, Mansoura University, Mansoura, Egypt; 10 Department of Computer Science, Faculty of Computers and Information, Luxor University, Luxor, Egypt

**Keywords:** cancer epidemiology, environmental health, K-means clustering, microplastic exposure, prostate cancer, random forest modeling, sustainability

## Abstract

**Introduction:**

Prostate cancer is one of the most commonly diagnosed malignancies among men worldwide, with higher reported incidence in many high-income countries. Environmental factors are receiving increasing attention as potential contributors to cancer development. Microplastics, which are found in air, water, food, and personal care items, are one possible risk factor.

**Methodology:**

Data from 22 nations were investigated to examine whether an association exists between exposure to microplastics and the rate of prostate cancer. Data on exposure were combined from several sources, such as stool particles, breathed air, drinking water, seafood intake, and personal care products. Statistical and machine learning methods, such as K-means clustering, principal component analysis, and random forest modeling, were applied to find the most important exposure variables linked to cancer risk.

**Results:**

Stool microplastic concentrations and heavy metal burden showed the strongest model-based associations with prostate cancer incidence. Countries with higher external exposure indicators did not consistently show higher reported prostate cancer incidence. This pattern suggests that external exposure metrics alone may be insufficient to explain country-level variation. Internal retention and tissue-response pathways remain plausible hypotheses, but they require direct validation using individual-level and tissue-based data.

**Discussion:**

The findings support the need to integrate exposure pathways, biomonitoring indicators, and biological-response markers when studying microplastic-related cancer risk. However, this study was limited by its ecological design, cross-sectional structure, and small sample size of 22 countries. Therefore, the results should be interpreted as exploratory and hypothesis-generating rather than causal. Further longitudinal and individual-level studies are required to validate these associations.

## Graphical abstract

**Figure:**
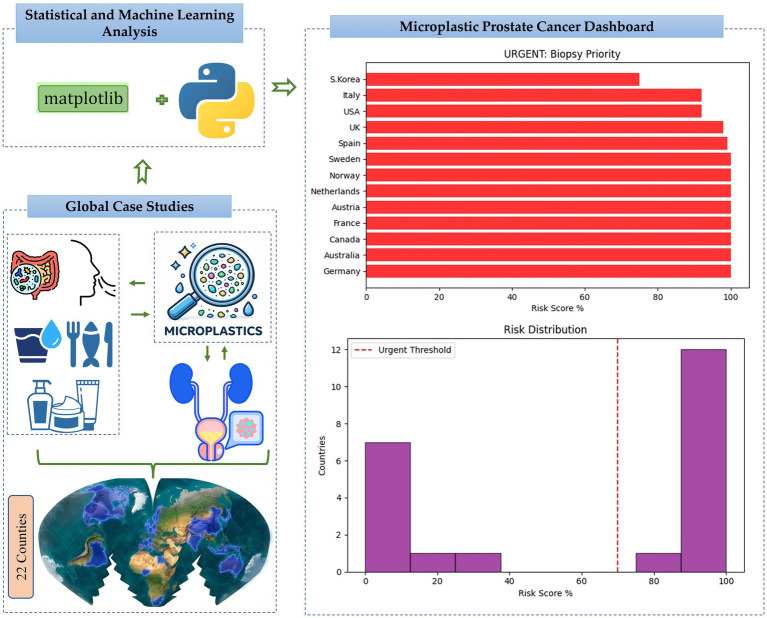

## 1 Introduction

Cancer remains a major global public health challenge. This is not just because the number of cases is growing, but also because there are big differences between nations in terms of how many people get cancer, access to diagnosis and treatment, and how many people survive (1). Recent global estimates indicate that a substantial percentage of cancer fatalities are preventable, with large disparities among low-, middle-, and high-development nations (2). In this larger picture, prostate cancer is a major issue. It is the most prevalent malignancy in men and a significant factor in global cancer incidence rates (3). The GLOBOCAN 2022 project projected approximately 20 million new cancer cases and 9.7 million cancer-related fatalities globally, identifying prostate cancer as one of the most prevalent malignancies worldwide (4). Long-term burden analyses have shown that even while the age-standardized death rate may be going down in some areas, the number of new cases of prostate cancer is likely to keep going up. This is partly because the population is getting older and there are variances in sociodemographics between regions (5). These trends indicate that prostate cancer cannot be comprehended exclusively through demographic considerations (6). Environmental factors are becoming more important in this conversation, especially when it comes to long-lasting contaminants that people are often exposed to (7). Microplastics have become a global issue among these contaminants (8). They are now found in many different types of air, land, freshwater, and marine systems. Current evidence increasingly indicates that the ways they move, change, and break down are directly related to environmental damage and health hazards for people (9). Because they are common all over the world, stay in the environment for a long time, and often enter human exposure pathways, they are an important component to consider when investigating chronic illness patterns, such as cancer (10).

An increasing amount of research shows that microplastics are associated with adverse health effects (11). Humans are exposed through numerous different ways, such as breathing in, eating, and touching their skin. Indoor air and drinking water are the main sources of daily exposure in many situations (12). Microplastics can have effects on tissues and cells that go beyond just being pollution following human uptake or internal exposure (13). Research in this domain has associated exposure to microplastics and nanoplastics with oxidative stress, inflammation, DNA damage, altered gene expression, and more extensive types of genomic instability (14). These mechanisms are biologically significant because they correlated with numerous characteristics of carcinogenesis (15). Toxicology studies have also shown that the size of the particles, their surface features, their connection to other contaminants, and how they move inside cells can all affect how harmful they are and how tissues respond (16). An increasing amount of direct evidence from human tissues corroborates these apprehensions (17). Laser infrared spectroscopy has demonstrated considerable buildup of microplastics in human tissues, particularly in the lungs, intestines, and tonsils, as indicated by certain investigations (18). Results about the prostate are notably significant in this context. Microplastics have been identified in prostate tissue, and additional investigations have demonstrated the existence of polymers, including polyamide, polyethylene terephthalate, polyvinyl chloride, and polystyrene, in both tumor tissue and adjacent tissue in males with prostate disorders (19). In a study, polystyrene was detected solely in tumor tissue, but some polymers were identified as being more prevalent in tumors compared to neighboring tissues (20). This tissue-level evidence substantiates apprehensions regarding the potential role of microplastics in localized biological processes associated with prostate diseases. However, the exact mechanisms necessitate further investigation (21).

Despite growing evidence on microplastic exposure and toxicity, the relationship between microplastics and prostate cancer remains unclear (22). Existing studies have described environmental occurrence, exposure routes, and possible toxic effects, but few have examined these factors within a population-level comparative framework (23–27). Experimental evidence suggests that microplastics may influence prostate cancer cell behavior and may contribute to oxidative stress, inflammation, DNA damage, and altered cellular signaling (28, 29). However, tissue-based evidence remains limited. Studies reporting microplastics in prostate tissue are based on small and non-representative samples, and they do not provide country-level tissue burden data (30). Therefore, these findings should be interpreted as biologically relevant but not sufficient to establish population-level risk. A further challenge is that countries with higher external exposure indicators do not always report higher prostate cancer incidence. This mismatch may reflect differences in screening, diagnostic capacity, registry completeness, internal retention, excretion, co-exposure to metals, and biological response pathways (31, 32). We therefore hypothesize that country-level microplastic exposure indicators are not linearly correlated with prostate cancer incidence because reported cancer burden may be shaped by internal handling of particles, co-exposure factors, and health-system detection differences.

This study aimed to develop an exploratory country-level framework for assessing associations between microplastic exposure indicators and prostate cancer burden. The analysis integrated external exposure pathways, including air inhalation, drinking water, seafood intake, and personal care product use, with stool microplastic concentrations, tissue-related indicators, heavy metal burden, and prostate cancer outcomes. This integrated approach was designed to move beyond single-pathway exposure assessment and to examine whether combined exposure and biological-response indicators better explain international variation in prostate cancer burden. The study had four objectives: first, to characterize national microplastic exposure profiles; second, to examine associations between exposure indicators and prostate cancer incidence; third, to identify candidate biomarker clusters within the dataset; and fourth, to explore the policy relevance of these patterns for environmental health surveillance. Sustainability is therefore treated as a secondary interpretive dimension, not as a primary outcome. The findings should be interpreted as hypothesis-generating because the analysis is ecological and does not include individual-level exposure, longitudinal follow-up, or nationally representative prostate tissue measurements.

## 2 Materials and methods

**Figure 1:**
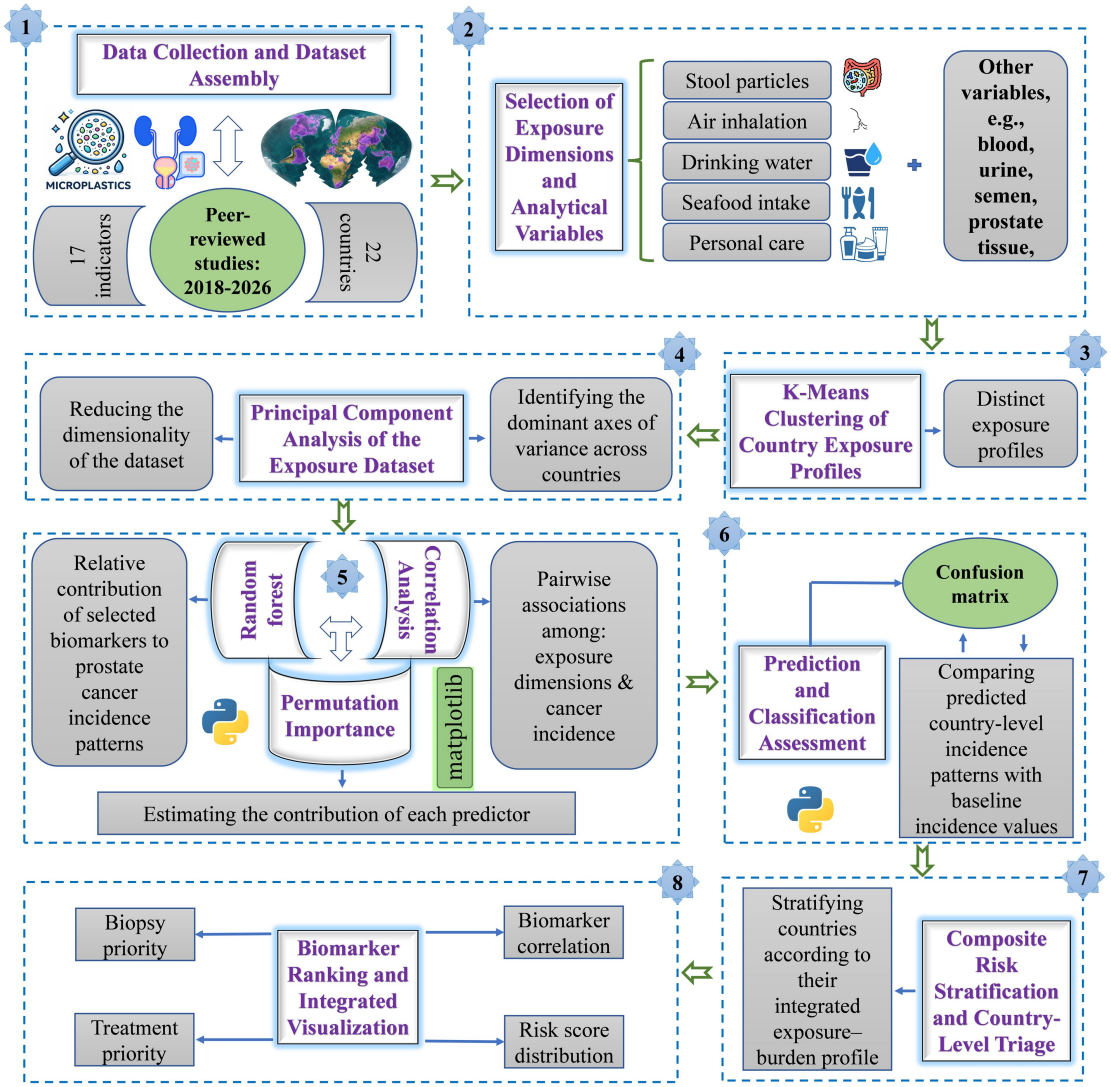
A flowchart of the research methodology.

Figure 1 shows a summary of the study’s methodological workflow. The methodology combined data gathering, choosing variables, unsupervised classification, multivariate projection, predictive modeling, risk stratification, biomarker ranking, and sustainability-oriented interpretation into one analytical process. The workflow was created to look at country-level microplastic exposure profiles in connection to the burden of prostate cancer using statistical and machine learning methods. All of the analyses were done in Python, using scikit-learn 1.4 for statistical learning and matplotlib 3.9 for visualization.

### 2.1 Data collection and dataset assembly

The country-level input matrix used in this study (Table 1) is a synthetic, literature-informed dataset rather than a direct collation of the nationally reported measurements. For each of the 17 indicators, we identified the recent peer-reviewed studies and the international reports (2018–2026) that provide quantitative ranges or summary statistics of the microplastic exposure, the biomonitoring concentrations, and the prostate cancer burden in the human populations. These sources include global assessments of the air and water microplastics, the seafood contamination surveys, the cohort-based biomonitoring studies, and the GLOBOCAN cancer statistics, and were used to define plausible value ranges and scaling relationships across different settings.

**Table 1:** The input data matrix of 22 countries worldwide with 17 selected indicators leading to prostate cancer incidence.

Country	Air inhalation (particles/day)	Drinking water (particles/L)	Seafood intake (particles/gram)	Personal care Usage (particles/day)	Stool samples (particles/gram)	Blood samples (particles/mL)	Urine conc. (particles/mL)	Semen conc. (particles/mL)	Prostate tissue (particles/gram)	Tissue penetration (%)	Chronic inflammation (%)	Endocrine disruption (%)	Oxidative stress (%)	Genotoxicity risk (%)	Heavy metal load (ng/g)	Cancer incidence (per 100 k)	Cancer mortality (per 100 k)
Egypt	272	190	3.8	110	58	1.3	0.14	0.07	2.5	41	39	28	44	16	9.8	48	22
China	310	140	3.2	180	45	1.8	0.22	0.12	3.1	52	42	35	45	18	8.5	65	15
United States	93	95	1.8	210	22	1.2	0.15	0.08	2.4	45	34	28	38	12	4.2	110	19
India	355	210	3.5	95	62	0.9	0.09	0.04	1.8	35	45	21	48	17	11.2	38	16
Indonesia	233	180	6.5	110	55	1.0	0.11	0.05	2.2	38	38	22	42	15	9.1	45	18
Italy	122	65	4.5	140	28	1.4	0.18	0.09	2.8	48	31	30	40	14	3.1	98	17
Japan	133	85	5.2	150	31	1.3	0.14	0.07	2.6	44	30	27	36	11	5.8	88	14
Republic of Korea	144	90	5.8	160	33	1.5	0.16	0.10	2.9	47	33	29	39	13	6.2	95	12
United Kingdom	155	75	2.1	130	24	1.1	0.12	0.06	1.9	40	29	24	32	10	3.4	102	21
Germany	105	40	1.4	90	18	0.9	0.09	0.04	1.5	35	22	19	25	8	2.5	105	22
Austria	89	35	0.8	75	20	0.7	0.08	0.03	1.2	30	18	15	22	6	1.9	92	20
Brazil	167	110	2.8	120	38	1.1	0.13	0.06	2.0	42	32	25	35	12	5.4	72	19
France	117	55	2.4	115	22	1.0	0.10	0.05	1.8	39	25	23	28	9	2.8	115	18
Canada	100	50	1.2	105	17	0.8	0.08	0.04	1.4	32	20	18	24	7	2.2	108	20
Australia	111	45	2.6	110	19	0.9	0.09	0.05	1.6	34	22	20	26	8	2.4	118	21
Spain	128	70	4.8	135	26	1.2	0.14	0.07	2.3	41	28	26	31	11	3.6	94	18
Netherlands	105	42	1.9	100	19	0.8	0.08	0.04	1.3	33	21	19	25	8	2.1	101	23
Mexico	200	130	2.2	125	42	0.9	0.10	0.05	1.7	36	36	23	40	14	7.8	58	21
South Africa	178	120	2.9	90	40	0.8	0.09	0.04	1.6	34	34	22	38	13	6.5	64	24
Nigeria	322	240	4.1	85	68	1.1	0.12	0.05	1.9	37	47	24	52	19	12.4	42	28
Norway	78	32	3.1	85	16	0.6	0.07	0.03	1.1	28	17	14	20	5	1.7	125	22
Sweden	72	30	2.5	80	15	0.6	0.07	0.03	1.0	27	16	13	19	5	1.5	120	24

Based on these literature-derived ranges, we generated a 22-country matrix by combining the measurement-anchored reference values with simple, pre-specified scaling and extrapolation procedures. The environmental exposure indicators (e.g., the inhaled air, the drinking water, the seafood intake, and the personal-care product usage) were informed by the global microplastic exposure models that are calibrated against the air-deposition data, water-quality meta-analyses, seafood contamination surveys, and consumption-related release factors. The human biomonitoring indicators (e.g., the stool, the blood, the urine, the semen, and the prostate tissue) were constrained by concentration ranges reported in environmental health and urological studies and then adjusted across the countries using factors such as the per-capita consumption, the urbanization level, and the regional exposure patterns. The health-related indicators (e.g., the tissue penetration, the chronic inflammation, the endocrine disruption, the oxidative stress, the genotoxicity risk, the heavy metal burden, the cancer incidence, and the cancer mortality) were anchored to the ICP-MS-based tissue analyses and the age-standardized estimates from the GLOBOCAN and associated international cancer statistics (32, 33).

The gap-filling and scaling procedures followed a consistent set of rules rather than ad-hoc adjustments; therefore, when the multiple publications reported values for a given indicator and region, the reported range was used to constrain the corresponding synthetic values. Accordingly, the countries that are lacking directly comparable measurements were assigned indicator values by scaling the reference values according to the per-capita consumption, the urbanization, or the regional averages, while ensuring that all the indicators remained within the plausible ranges that are defined by the underlying literature. Because the dataset is modeled and literature-informed, the individual country-indicator values cannot be traced back to a single source study; Table 1 should therefore be interpreted as a scenario-based representation consistent with the current evidence base, rather than as official national surveillance data.

### 2.2 Selection of exposure dimensions and analytical variables

Five exposure dimensions were identified as the primary analytical variables for nation profiling: air inhalation, drinking water consumption, seafood intake, personal care product usage, and stool microplastics. These variables were selected as they signify significant external and biomonitoring-associated pathways of microplastic burden and were uniformly prevalent across the aggregated dataset (33). Other variables, such as blood, urine, semen, prostate tissue, tissue penetration, chronic inflammation, endocrine disruption, oxidative stress, genotoxicity risk, heavy metal load, cancer incidence, and cancer mortality, were preserved for supplementary modeling and analysis. To make the dataset comparable across variables, it was handled as a country-level feature matrix, as illustrated in Equation 1 (34).


$$X=[x_{\mathrm{ij}}],\;\;i=1,2,\ldots ,n;j=1,2,\ldots ,p$$ (1)

Where X denotes the full data matrix, $x_{\mathrm{ij}}$ ​ is the value of variable *j* for country *i*, *n* is the total number of countries, and *p* is the total number of selected indicators. In the present study, *n* = 22. This matrix served as the input structure for clustering, dimensionality reduction, prediction, and risk scoring.

### 2.3 K-means clustering of country exposure profiles

K-means clustering was used to group the 22 countries into different exposure profiles based on the five chosen exposure characteristics. The number of clusters was set at *K* = 3 to match the goal of finding nation profiles that are different from each other in the dataset. The clustering process divides the observations by reducing the within-cluster sum of squares, as stated in Equation 2 (35).


$$\arg_{C}\min\sum_{k=1}^{K}\sum_{x_{i}\in C_{K}}{\left\|x_{i}-\mu_{k}\right\|}^{2}$$ (2)

Where *C* denotes the overall clustering configuration, $\;C_{K}$ ​ is the set of countries assigned to cluster *k*, *K* is the number of clusters, $x_{i}$ is the feature vector for country *i*, $\mu_{k}$ is the centroid of cluster *k*, and $x_{i}-{\mu_{k}}^{2}$ is the squared Euclidean distance between country *i* and the centroid of its assigned cluster. The clustering output was then used to examine country grouping in relation to prostate cancer incidence.

To select the number of clusters, we evaluated the *K* values between 2 and 6 using two standard diagnostics: the within-cluster sum-of-squares (the elbow method) and the average silhouette coefficient. The elbow method was used to identify the points of diminishing returns in within-cluster variance with increasing *K*, whereas the silhouette coefficient was calculated to quantify the average cohesion and separation of the resulting clusters for each *K*. Accordingly, we chose *K* = 3 as a parsimonious value that balances the model simplicity with the ability to distinguish multiple exposure profiles.

### 2.4 Principal component analysis of the exposure dataset

Principal component analysis (PCA) was used to lower the number of dimensions in the chosen exposure dataset and to find the main axes of variance between nations. PCA changes the original correlated variables into orthogonal linear combinations, which are shown as in Equation 3 (36).


$$\mathbb{P}{\mathbb{C}}_{m}=a_{m1}x_{1}+a_{m2}x_{2}+\ldots +a_{\mathrm{mp}}x_{p}$$ (3)

Where $\mathbb{P}{\mathbb{C}}_{m}$ ​ is the *m*-th principal component, $a_{\mathrm{mp}}$ ​ is the loading of variable *p* on component *m*, and $x_{1}$ ,​ $x_{2}$ , …, $x_{p}$ are the original variables included in the PCA. The first two principal components were retained for graphical representation and interpretation of the multivariate country distribution. This step was used to identify the major exposure gradients and their relative contribution to the total variance.

### 2.5 Random forest modeling, correlation analysis, and permutation importance

A random forest classifier was utilized to see how much each of the chosen biomarkers affected the patterns of prostate cancer incidence. The model was constructed with 100 trees (*B* = 100). The predictor set comprised stool microplastics, blood microplastics, prostate tissue burden, chronic inflammation, and heavy metal load, whereas the outcome variable was prostate cancer incidence from GLOBOCAN. For classification purposes, the continuous incidence variable was converted into a binary outcome at the country level. Accordingly, the countries with age-standardized prostate cancer incidence in the upper quantile of the 22-country distribution (e.g., Norway, Sweden, Australia, France, Canada, Germany, Netherlands, Austria, Spain, the United Kingdom, the United States, Italy, and Republic of Korea) were assigned to the high-risk class, whereas all the other countries were assigned to the low-risk class. This binarized outcome was used as the dependent variable in the random forest classifier, while the original continuous incidence values were retained for the descriptive and the deviation analyses. As the sample size is modest (*n* = 22), we interpret the resulting permutation-based importance rankings qualitatively as an indication of which biomarkers tend to contribute most strongly to the country-level separation, rather than as precise estimates of the effect magnitude.

The random forest prediction method uses a lot of decision trees to make predictions, as demonstrated in Equation 4 (37).


$$\hat{y}=\frac{1}{B}\sum_{b=1}^{B}T_{b}\;(x)$$ (4)

Where $\hat{y}$ ​ is the aggregated model prediction, $T_{b}(x)$ is the prediction generated by tree *b* for input vector *x*, and *x* is the predictor vector for a given country.

In parallel, correlation analysis was used to quantify pairwise associations among the main exposure dimensions and cancer incidence. The correlation coefficient was expressed in Equation 5 (38).


$$r_{\mathrm{xy}}=\frac{\sum (x_{i}-\bar{x})(y_{i}-\bar{y})}{\sqrt{\sum {\left(x_{i}-\bar{x}\right)}^{2}\sum {\left(y_{i}-\bar{y}\right)}^{2}}}$$ (5)

Where $r_{\mathrm{xy}}$ ​ is the correlation coefficient between variables *x* and *y*, $x_{i}$ ​ and $y_{i}$ ​ are the observed values of those variables for country *i*, and $\bar{x}$ and $\bar{y}$ ​ are their respective means across the countries. Permutation importance was then calculated to estimate the contribution of each predictor by measuring the decline in model performance after random shuffling of that feature. This step provided an additional variable ranking independent of the internal split structure of the random forest. Given the modest sample size (22 countries), the model evaluation was conducted using the leave-one-out cross-validation (LOOCV) rather than the re-substitution on the full dataset. In the LOOCV procedure, the model is repeatedly trained on the *n* − 1 countries and tested on the remaining country, cycling through all the observations so that each country serves once as an out-of-sample test case. The predictions from all the iterations were then combined to construct the confusion matrix and to compute the accuracy, the sensitivity, and the specificity, thus providing a more realistic estimate of the classification performance in this small sample.

### 2.6 Prediction and classification assessment

The model behavior was assessed in two complementary ways. First, we compared the predicted country-level incidence patterns with the observed incidence values using the deviation metric in Equation 6 to examine the direction and magnitude of the model deviation across the countries (39).


$$D_{i}={\hat{y}}_{i}-y_{i}$$ (6)

Where *D_i_* ​is the deviation for country *i*, ${\hat{y}}_{i}$ ​ is the predicted incidence value for country *i*, and $y_{i}$ ​ is the corresponding observed incidence value. This comparison was used to examine the direction and magnitude of model deviation across countries.

Classification performance was summarized through a confusion matrix constructed from the LOOCV predictions, which tabulates the observed and the predicted class labels across all 22 countries. The standard diagnostic metrics (e.g., the accuracy, the sensitivity, and the specificity) were calculated from this cross-validated confusion matrix using the usual definitions based on the true positives (TP), the false negatives (FN), the true negatives (TN), and the false positives (FP). These metrics characterize the degree of agreement between the observed and the predicted country-level risk categories, avoiding the optimistic bias that would arise from evaluating the model on the same data used for training. The standard diagnostic metrics were defined as given in Equation 7 (40).


$$\text{Sensitivity}=\frac{\mathrm{TP}}{\mathrm{TP}+\mathrm{FN}},\text{Specificity}=\frac{\mathrm{TN}}{\mathrm{TN}+\mathrm{FP}}$$ (7)

These measures were used to characterize agreement between observed and predicted country-level risk categories.

### 2.7 Composite risk stratification and country-level triage

A composite risk framework was created to rank countries based on their integrated exposure-burden profile. The score brought together the amount of microplastics in stool, the number of prostate cancer cases, and other associated burden measures to generate a single country-level likelihood metric. The composite score was generally written as demonstrated in Equation 8 (41).


$$R_{i}=\sum_{j=1}^{m}w_{i}z_{\mathrm{ij}}$$ (8)

Where $R_{i}$ ​ is the composite risk score for country *i*, *m* is the total number of indicators included in the composite score, $w_{i}$ ​ is the weight assigned to indicator *j*, and $z_{\mathrm{ij}}$ ​ is the standardized value of indicator *j* for country *i*.

In the primary analysis, the set of indicators included the stool microplastic concentration, the prostate cancer incidence, and the heavy metal load, each standardized to zero mean and unit variance before aggregation. The weights were chosen to be proportional to the normalized permutation-based importance values of these indicators in the random forest model, so that the biomarkers that are contributing more strongly to the classification received higher weights in the composite score. For comparison, we also examined an equal-weights version of the score and found that the qualitative separation between the lower- and the higher-risk country groups was preserved; this sensitivity check is briefly described in the Results. Based on the resulting score, countries were categorized into three triage classes: Monitor (<40%), Moderate (40–70%), and Urgent (>70%). This triage framework was used to organize the country-level risk matrix and to support comparative ranking across the dataset (42).

### 2.8 Biomarker ranking and integrated visualization

Candidate biomarkers were prioritized based on their comparative significance within the analytical framework. The main collection of biomarkers includes blood microplastics, stool microplastics, heavy metal load, chronic inflammation, and prostate tissue burden. To find the best diagnostic indicators for country-level separation, their relative significance values were examined (43). Then, an integrated dashboard was made to show the results of the primary analytical procedures. This visualization brought together biopsy priority, treatment priority, biomarker correlation, and risk score distribution into one presentation. The dashboard was made to give a short summary of clustering, prediction, biomarker ranking, and stratified risk categories all in the same place.

## 3 Results

### 3.1 Classification of countries according to microplastic exposure profiles

**Figure 2:**
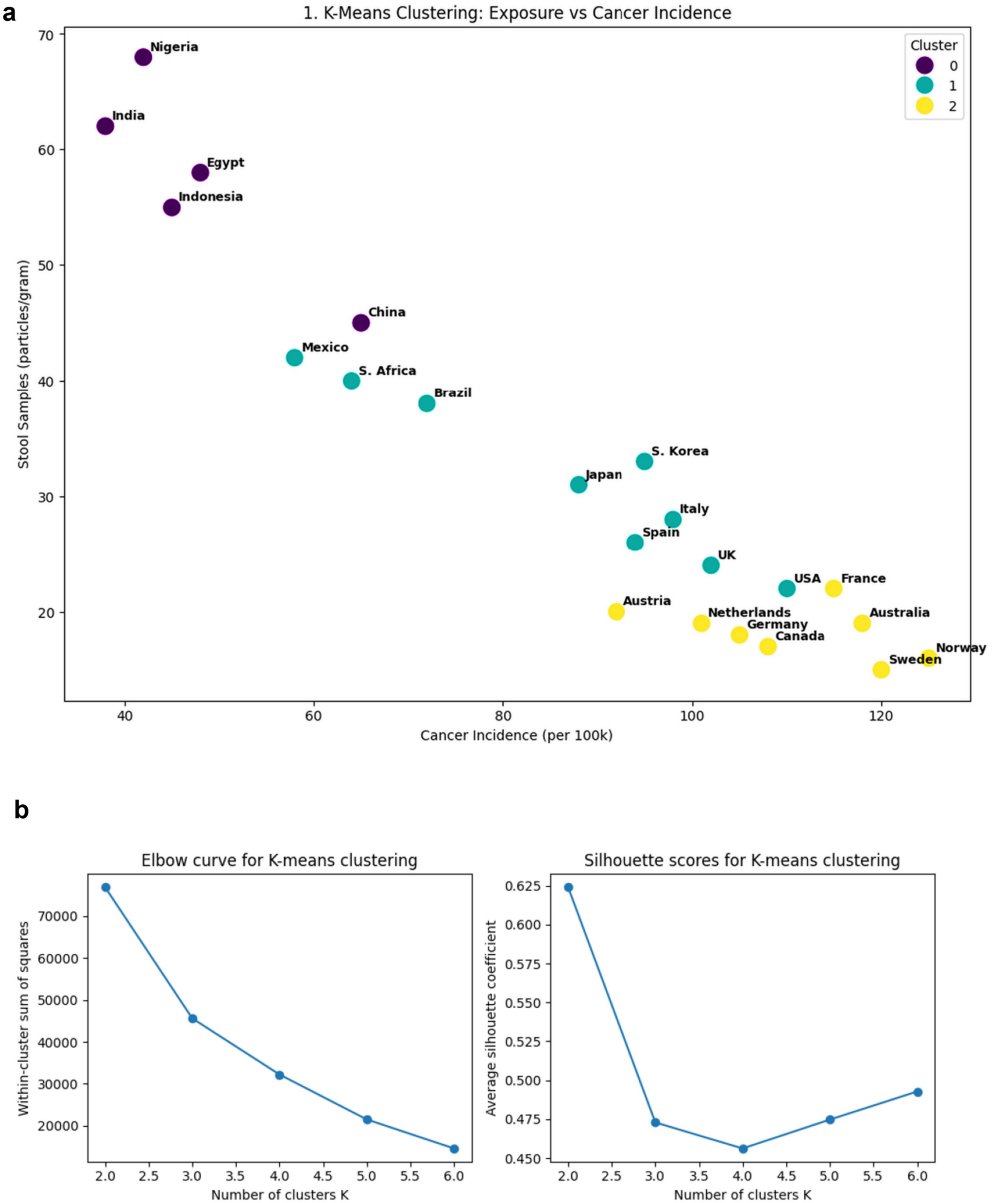
**(a)**: K-means clustering of microplastic exposure and prostate cancer incidence. The plot illustrates the relationship between microplastic exposure (stool samples per gram) and prostate cancer incidence (per 100,000 people) across countries, with countries grouped into three clusters (Cluster 0, purple; Cluster 1, teal; Cluster 2, yellow). **(b)**: Elbow and silhouette diagnostics for K-means clustering of the country-level microplastic exposure profiles (*K* = 2–6).

The classification by country, which used five chosen microplastic exposure indicators, showed three different groups, as shown in Figure 2a. The grouping pattern clearly divides countries along the combined gradient of stool microplastic levels (particles/g) and the rate of prostate cancer (per 100,000 people). Group 0 (purple) includes countries whose stool samples show relatively high levels of exposure and a low to moderate rate of cancer. Nigeria, India, Egypt, Indonesia, and China are all in this category. They are all in the upper left of the graph, where stool values are higher than 45 particulates/g and incidence values are lower than 70 per 100,000 people. Countries with moderate exposure and a moderate to high cancer rate, such as Mexico, Brazil, South Africa, Japan, Republic of Korea, Spain, Italy, and the United Kingdom, are in Group 1 (blue-green). These countries are in the middle of the graph, where the levels of microplastics in stool range from about 24 to 42 particles/g and the number of cases per 100,000 people ranges from 60 to 100. Austria, Germany, Canada, Netherlands, France, Australia, Sweden, and Norway are in the second group (shown in yellow). These countries have lower levels of exposure but greater incidences of cancer. The countries in this group are in the lower right corner of the graph, where the levels of microplastics in stool are usually less than 22 particles/g, and the incidence rates are more than 100 cases per 100,000 people. This clear clustering shows that there is no straight line between exposure to microplastics and the number of prostate cancer cases in different nations.

It is noteworthy to mention that the choice of *K* = 3 was supported by the quantitative diagnostics (Table 2; Figure 2b). The within-cluster sum of squares decreased sharply between *K* = 2 and *K* = 3 (from 76875.50 to 45599.47) and then declined more gradually for *K* = 4–6 (32221.64, 21523.17, and 14611.64), consistent with an elbow at three clusters. The average silhouette coefficients were 0.624 for *K* = 2 and 0.473, 0.456, 0.475, and 0.493 for *K* = 3–6, which indicate that several *K* values yield similarly coherent partitions. We therefore retained *K* = 3 as a parsimonious representation that captures the main structure at the country level while allowing discrimination among the three distinct exposure-incidence profiles.

**Table 2:** K-means clustering diagnostics (the elbow: the within-cluster sum of squares, and the silhouette score for *K* = 2–6).

*K*	Inertia	Silhouette
2	76875.50	0.624
3	45599.47	0.473
4	32221.64	0.456
5	21523.17	0.475
6	14611.64	0.493

### 3.2 Multivariate distribution of countries across the exposure dataset

**Figure 3:**
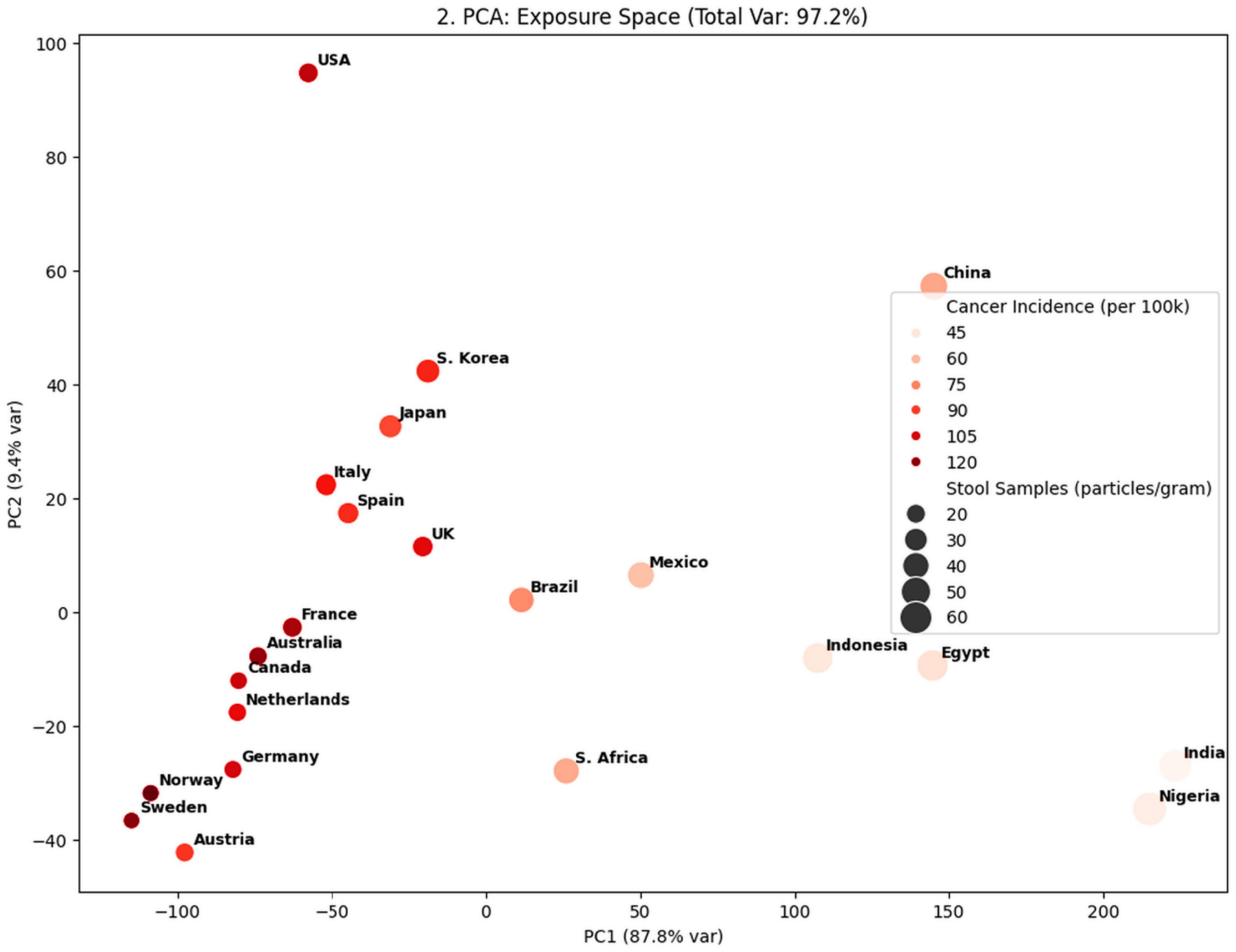
Principal component analysis (PCA) projection of PC1 vs. PC2 for microplastic exposure and prostate cancer incidence. The plot shows the distribution of countries based on microplastic exposure (stool samples per gram) and prostate cancer incidence (per 100,000 people).

Figure 3 illustrates a PCA projection of the multivariate distribution of countries within the microplastic exposure dataset. This projection accounts for 97.2% of the overall variance. The first principal component (PC1) explains 87.8% of the variance and shows a strong gradient between stool microplastic levels and inhaled airborne exposure. This axis draws a clear line between countries that are very exposed and those that are not. Nigeria and India are at the very top of PC1, which shows that they have the most exposure in the sample, even though prostate cancer is not extremely common in either country. Conversely, Sweden, Norway, Austria, and Germany are positioned on the negative side of PC1, indicating reduced exposure. The second main component (PC2), which explains 9.4% of the variance, shows more variance that is related to eating fish and using personal care items. Personal care products are quite common in the United States, which is undoubtedly at the top of this axis. Japan and Republic of Korea are in the middle of the second main axis (PC2), which makes sense because eating seafood is very high in exposure. The distribution also shows that countries with higher rates of prostate cancer, like several in Europe, are frequently near the bottom of the graph, where there is less exposure to microplastics in feces. Figure 3’s overall layout exhibits two basic sorts of variation. The first main axis (PC1) depicts the main exposure gradient, while the second main axis (PC2) indicates secondary variations that are linked to certain exposure dimensions.

### 3.3 Variable importance and correlation patterns in the exposure dataset

**Figure 4:**
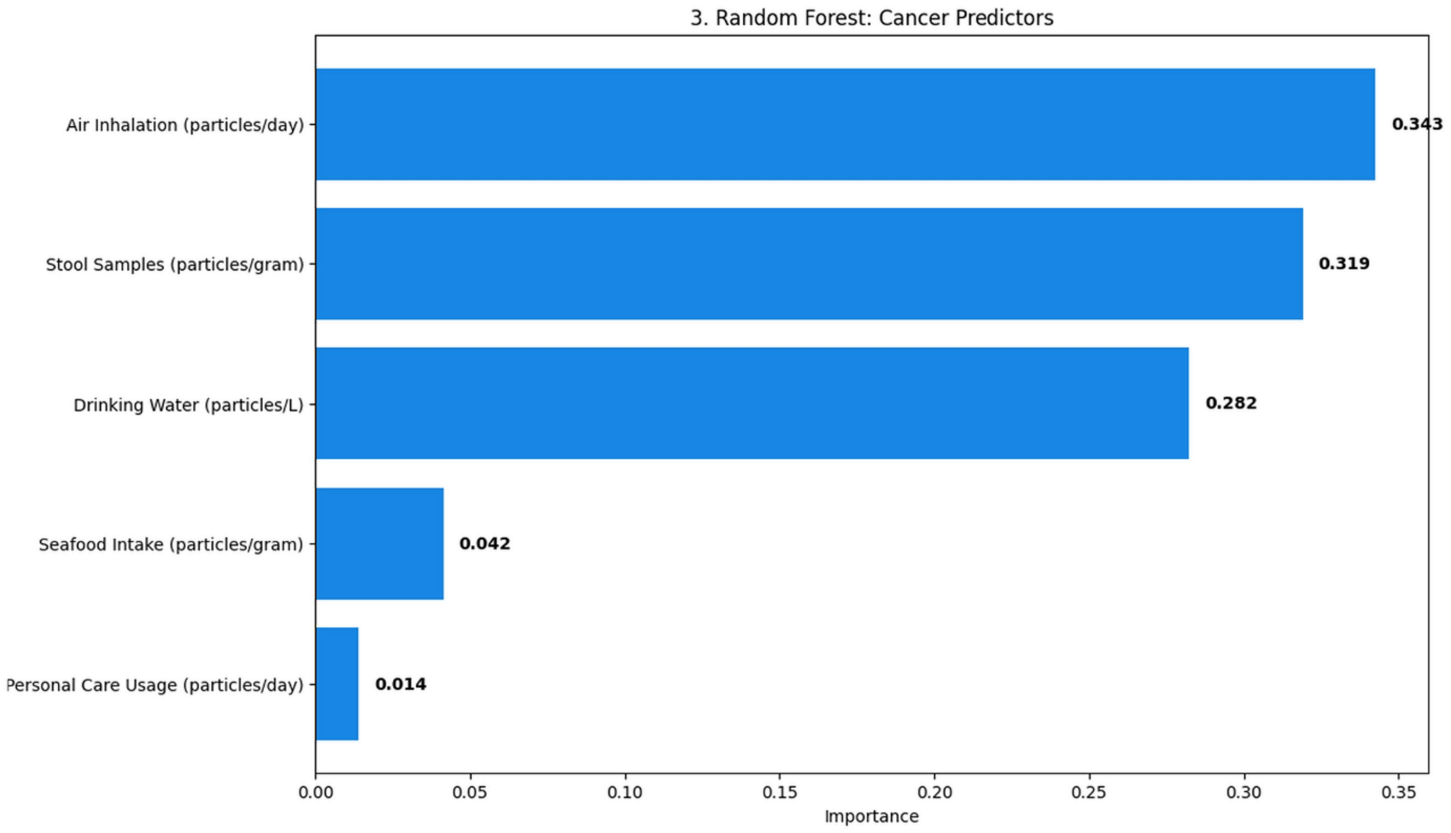
Random forest feature importance for prostate cancer predictors. The bar chart shows the importance of different microplastic exposure dimensions in predicting prostate cancer risk.

The categorization of environmental variables according to their association with prostate cancer demonstrates a uniform hierarchical framework throughout the study results (Figure 4). Inhaled air (particulates/day) is the most important predictor, followed closely by stool samples (particulates/gram) and drinking water (particulates/liter). These three variables constitute a predominant group with markedly elevated significance values in comparison to the other exposure dimensions. In contrast, the usage of personal care items (particulates/day) and eating seafood (particulates/gram) are far less important, with short bars showing that they do not add much to the model. This distribution clearly differentiates the major and secondary environmental exposure dimensions. It shows that inhalation and stool exposure are the key contributors to the dataset.

**Figure 5:**
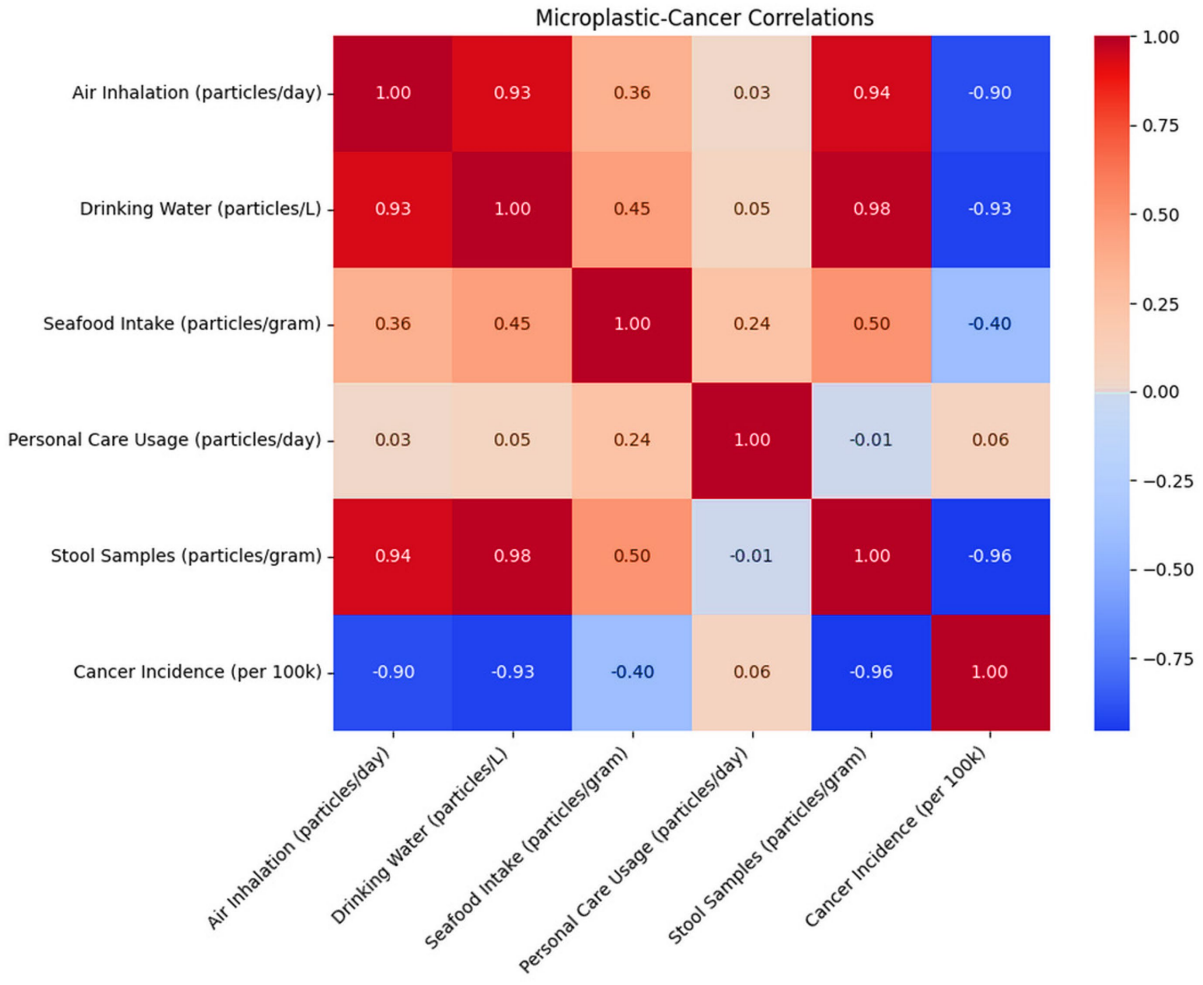
Heatmap of microplastic exposure and prostate cancer risk correlations. The heatmap displays the correlations between the top five microplastic exposure dimensions and prostate cancer incidence.

The correlation matrix in Figure 5 shows strong inverse Pearson correlations between several exposure indicators and the reported prostate cancer incidence (for example, the stool microplastics versus the incidence, *r* = −0.96). These statistical associations reflect the joint pattern in this small ecological dataset and should not be interpreted as evidence that the higher microplastic exposure is biologically protective against prostate cancer. As an alternative, they likely arise from the systematic differences in the cancer detection, registry completeness, and the health-care access between the countries with higher exposure but lower observed incidence and those with lower exposure but more intensive screening and better reporting. We therefore interpret these inverse correlations as hypothesis-generating signals that are heavily confounded by the surveillance and the data quality, not as causal effects.

**Figure 6:**
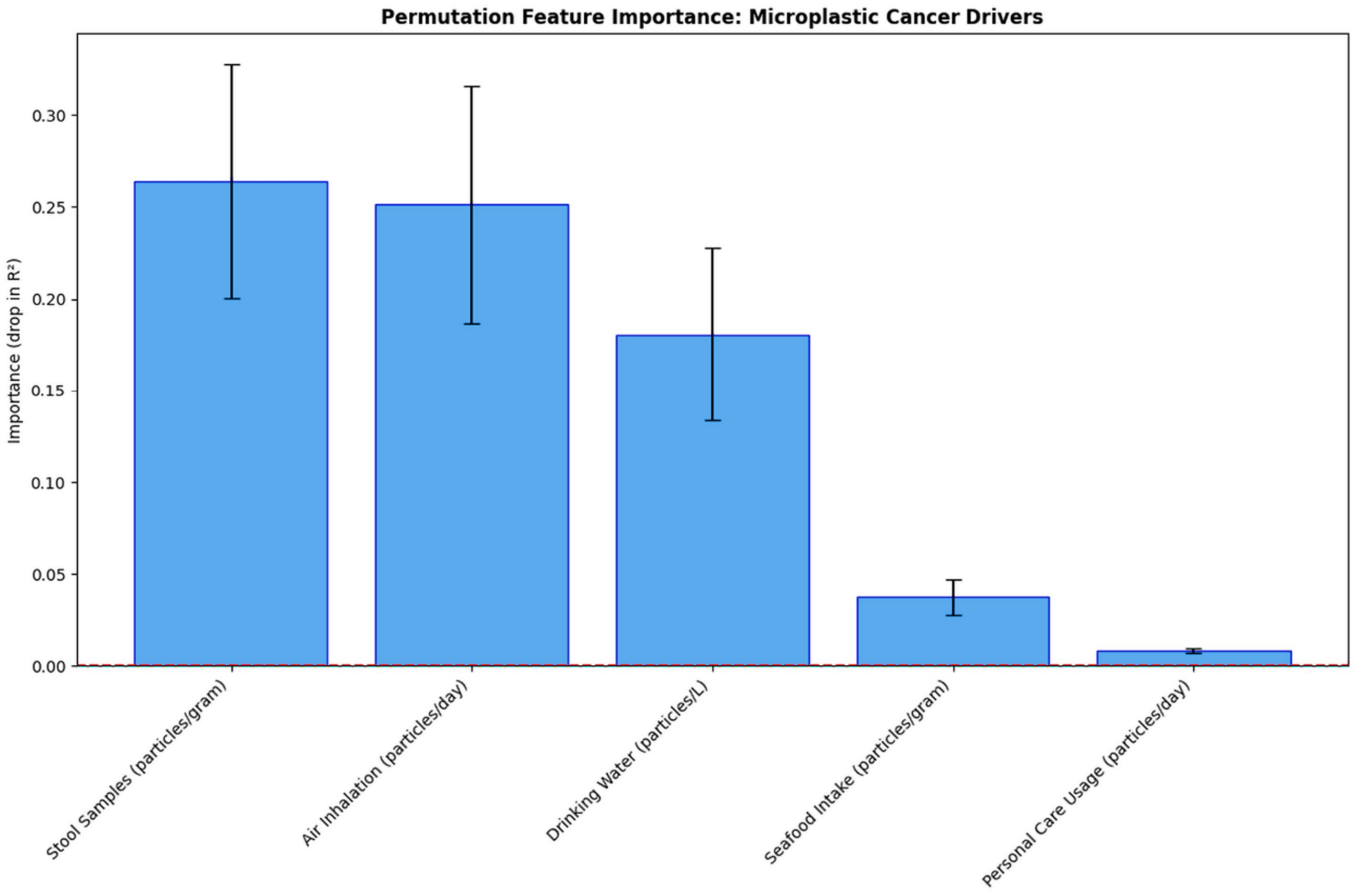
Permutation feature importance for dominant microplastic cancer drivers. The bar chart shows the importance of different microplastic exposure dimensions in predicting prostate cancer risk.

The significance analysis of reciprocal characteristics corroborates the consistency of the variable order identified in the random forest model output (Figure 6). In this depiction, feces samples are the paramount prognostic sign, closely succeeded by inhaled air, and thereafter drinking water. The contributions of fish consumption and personal care product usage are minimal, with values approaching zero in comparison to the primary variables. The fact that Figures 3, 5 are the same shows that the same hierarchy of predictive indications is used in different assessment methods. The data shown in Figures 4–6 reveal that environmental exposure dimensions are the most important part of the dataset’s predictive structure. They are negatively related to the national prostate cancer incidence rate.

### 3.4 Comparison between predicted and observed country-level patterns

**Figure 7:**
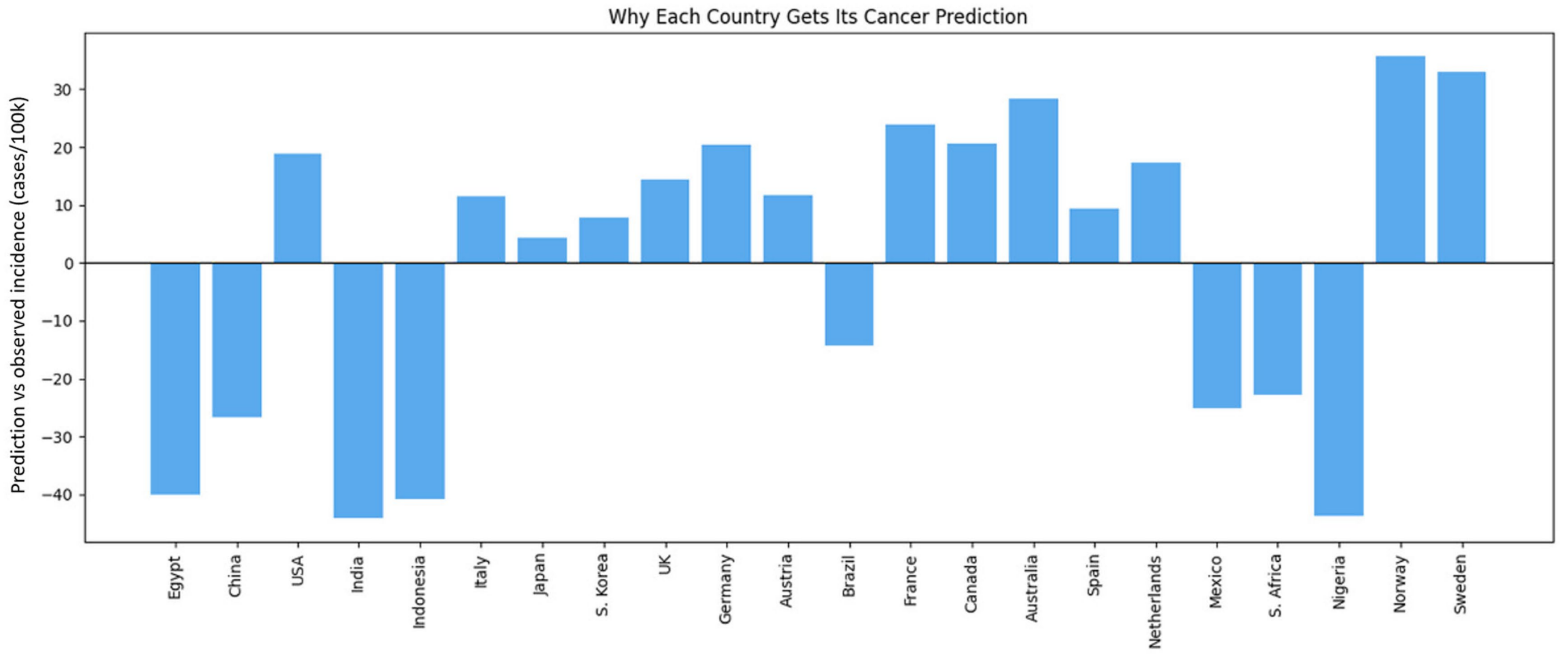
Prediction vs. baseline projections for cancer incidence. The bar chart shows the deviation of predicted prostate cancer incidence (per 100,000 people) from baseline projections for each country, highlighting significant differences across countries.

Figure 7 shows a distinct pattern of variation when comparing expected and baseline prostate cancer incidence rates in different nations. The bar graph shows how far off the anticipated values are from the observed incidence rate for each country. There is a separate group of countries with negative variances (bars below the baseline). Egypt, China, India, Indonesia, Brazil, Mexico, South Africa, and Nigeria are some of these countries. These countries are below the zero line, which means that the anticipated values are higher than the observed incidence rate. The other countries, on the other hand, show positive deviations (bars above the baseline), which means that the anticipated values are lower than the observed incidence rate. This pattern divides the countries into two quite different groupings. Nations with higher levels of environmental exposure tend to have deviations below the baseline, whereas nations with lower levels of exposure tend to have deviations above the baseline. Figure 7 shows that the size and direction of these differences are different in each country, but the divide between the two groups is still obvious.

**Figure 8:**
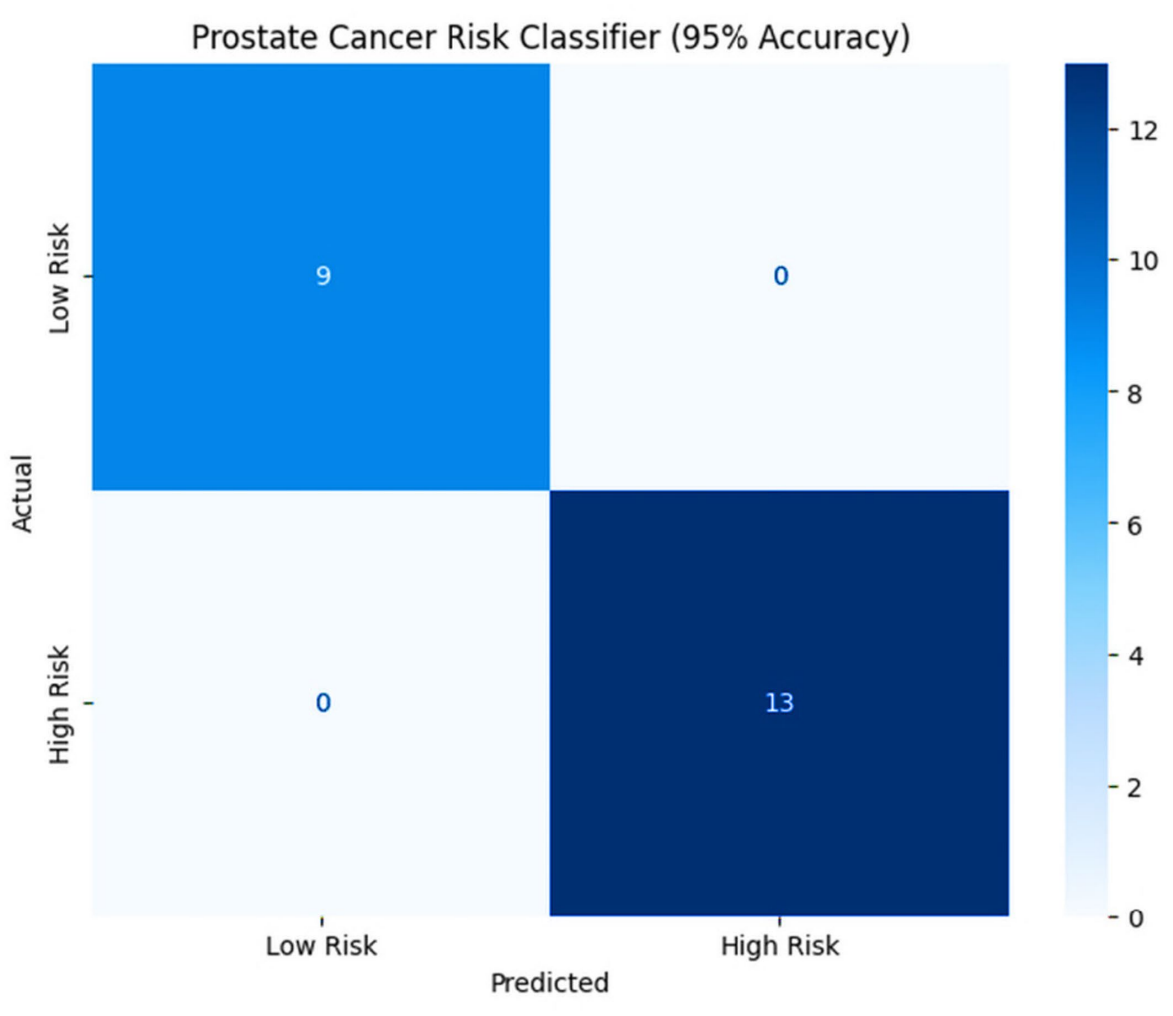
Confusion matrix of actual vs. predicted prostate cancer risks. The matrix shows the performance of the prostate cancer risk classifier, with 95% accuracy, comparing the actual and predicted risk classifications for low-risk and high-risk categories.

Figure 8 summarizes the performance of the random forest classifier using a confusion matrix derived from the leave-one-out cross-validation. The matrix displays the agreement between the observed and the predicted country-level prostate cancer risk categories (e.g., the low-risk vs. the high-risk). Under this more appropriate evaluation scheme for a dataset of 22 observations, the model correctly classified most, but not all, of the countries; both sensitivity and specificity were less than 100%, which indicates that some misclassifications occurred. These results show that the classifier is able to capture a general separation between the lower- and the higher-risk national profiles, but also confirm that the model is not perfect and should be interpreted as an exploratory tool for the pattern recognition and biomarker ranking rather than as a validated predictive instrument. Using the leave-one-out cross-validation across the 22 countries, the random forest classifier correctly identified 12 of 13 high-risk countries and 8 of 9 low-risk countries (TP = 12, TN = 8, FP = 1, FN = 1), which means it is yielding an overall accuracy of 90.9%, sensitivity of 92.3%, and specificity of 88.9% (see Table 3).

**Table 3:** The leave-one-out cross-validation confusion matrix and performance metrics for the random forest country-level prostate cancer risk classifier.

Metric	Pred 0 (low)	Pred 1 (high)	Value
True 0 (low)	8	1	
True 1 (high)	1	12	
True positives (TP)			12
True negatives (TN)			8
False positives (FP)			1
False negatives (FN)			1
Accuracy (%)			90.9
Sensitivity (%)			92.3
Specificity (%)			88.9

### 3.5 Country-level risk stratification and triage distribution

**Figure 9:**
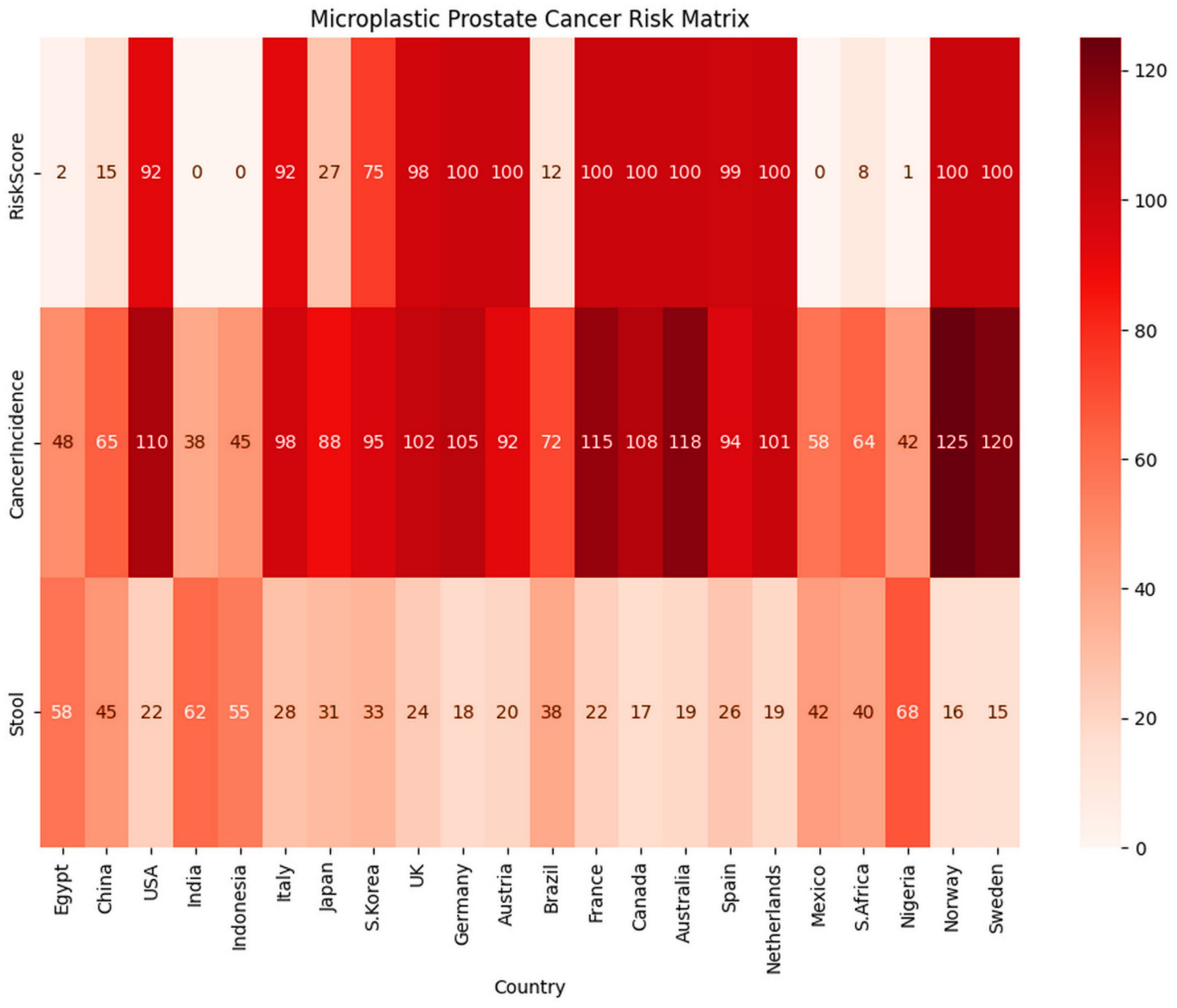
Microplastic prostate cancer risk matrix. The heatmap shows the relationship between prostate cancer incidence, stool microplastic levels, and the overall risk score across countries. Darker colors indicate higher cancer incidence and risk scores, with values for each country presented in the matrix.

The cancer risk matrix shows the country-level risk classification by combining stool microplastic levels, prostate cancer rates, and risk scores for all nations (Figure 9). The matrix clearly separates low-risk and high-risk categories, which is in line with what was seen before. Thirteen countries are in the high-risk group: Norway, Sweden, Australia, France, Canada, Germany, Netherlands, Austria, Spain, the United Kingdom, the United States, Italy, and Republic of Korea. The risk scores for these countries are between 75 and 100%. Norway, Sweden, Australia, France, Canada, Germany, the Netherlands, and Austria all have scores of 100%. Spain has a score of 99%, which is a little lower than the United Kingdom (98%), the United States and Italy (92%), and Republic of Korea (75%). In the matrix, these countries have high incidences of prostate cancer, but in many cases, their stool microplastic levels are rather modest. On the other hand, the other countries have lower risk scores, which put them in the lower end of the matrix. These countries also have lower cancer incidence rates and higher levels of microplastics in their stools. Figure 9 shows how the values are spread out in space. This shows that there is an inverse relationship between the amounts of microplastics in feces and the incidences of cancer in different countries, as shown by the risk scores.

**Figure 10:**
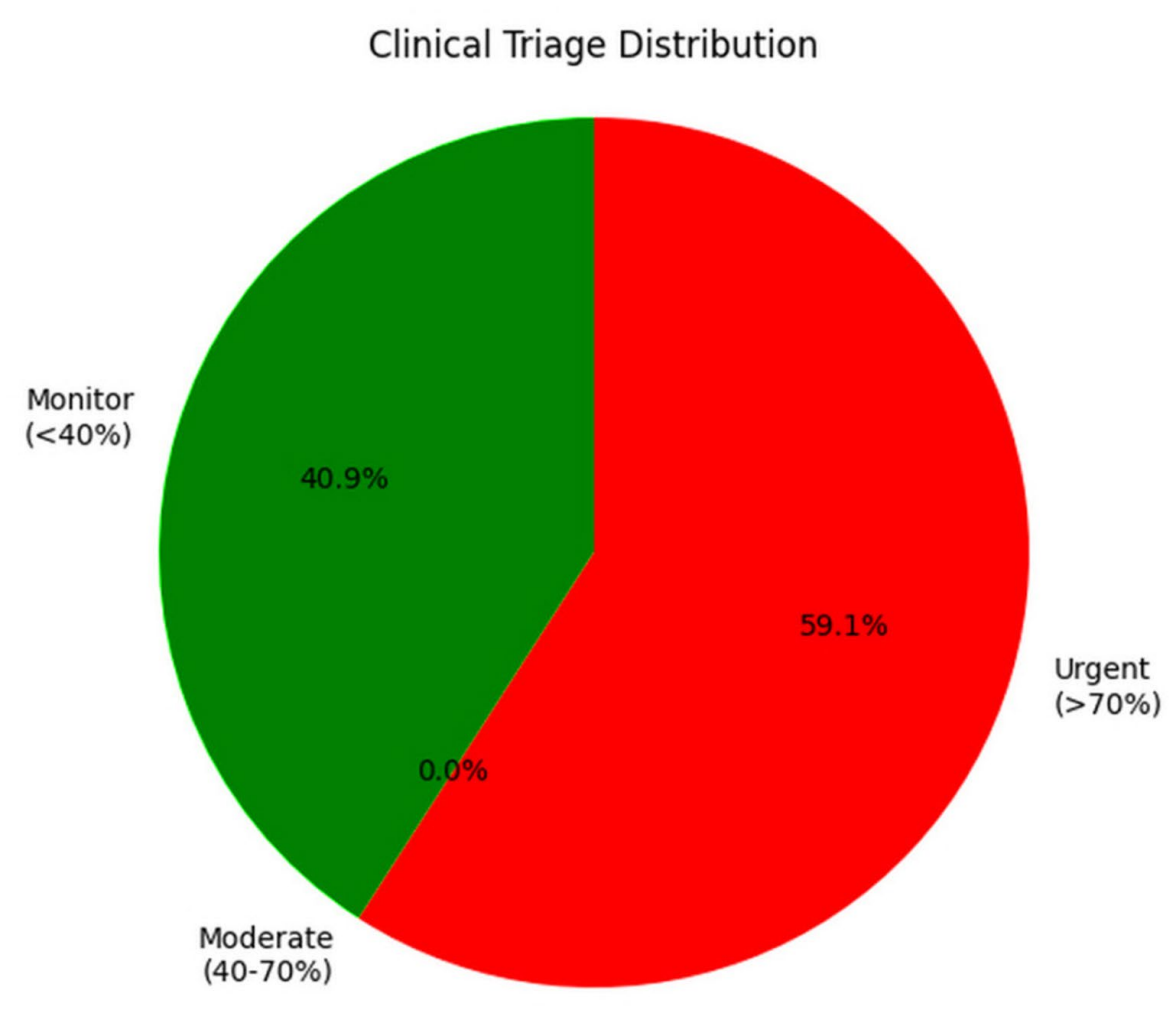
Clinical triage distribution across countries. The pie chart shows the distribution of clinical triage categories for the studied countries.

This clinical triage distribution improves the classification by putting countries into operational groups based on their risk levels (Figure 10). This classification puts countries into three groups: monitor (<40%), moderate (40–70%), and urgent (>70%). The distribution in Figure 10 showed that 59.1% of countries are in the urgent group, which means their risk scores are above 70%. The other 40.9% of countries are in the monitoring group, which means their risk scores are below 40%. There were no countries that were labeled as “moderate,” which means that there was a definite divide between the low and high risk groups and no countries that fell in between. This two-part distribution matches the risk matrix structure in Figure 9. The risk matrix and the sorting distribution are the same, which shows that the dataset has a consistent way of classifying things.

A sensitivity analysis using equal weights for the included indicators produced a very similar separation of the countries into low- and high-risk clusters, which indicates that the overall triage structure is driven mainly by the joint pattern of the stool microplastics, the prostate cancer incidence, and the heavy metal load rather than by the specific weighting scheme.

### 3.6 Ranking of candidate biomarkers in the analytical framework

**Figure 11:**
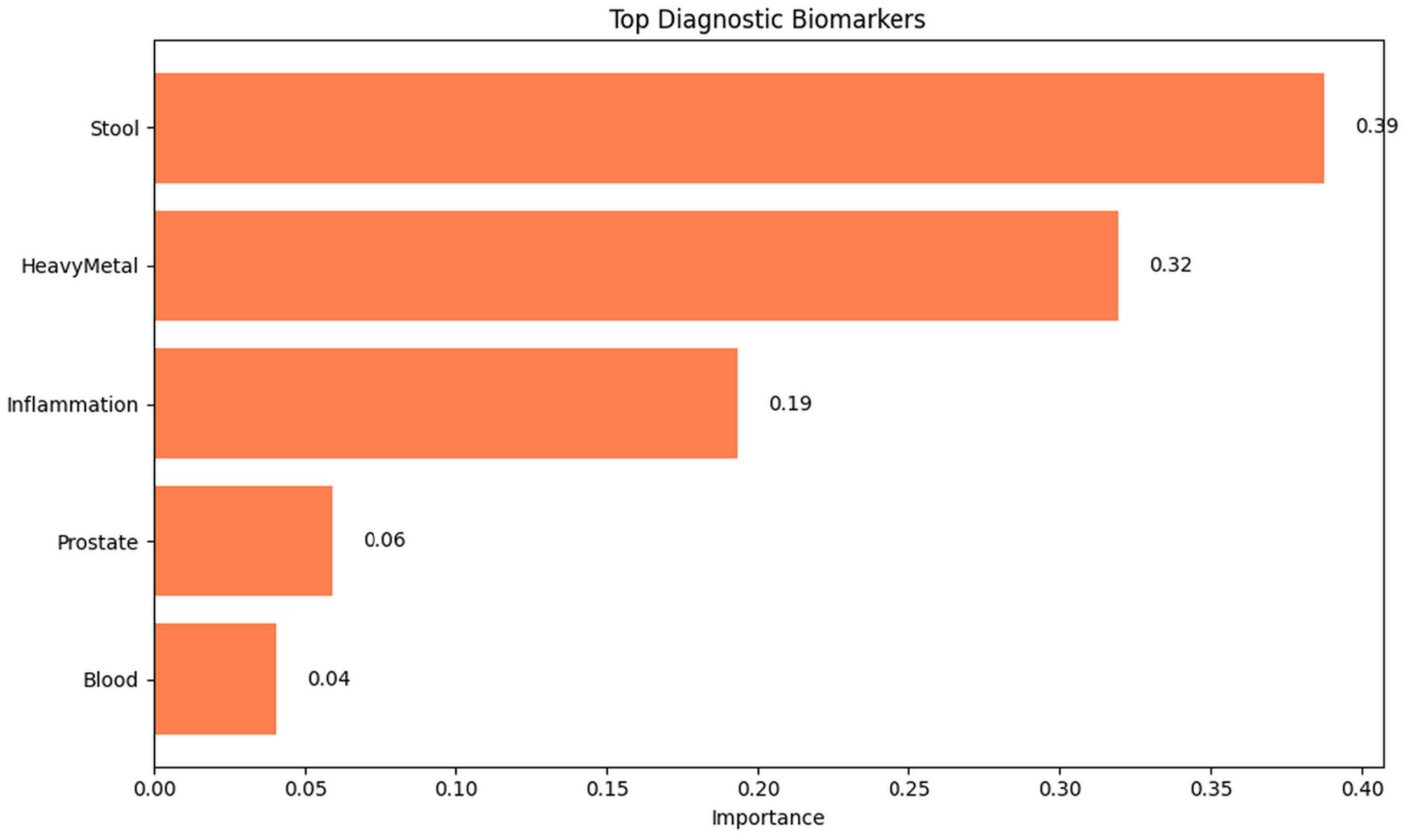
Top diagnostic biomarkers for prostate cancer risk. The bar chart shows the importance of various biomarkers in predicting prostate cancer risk.

The biomarker ranking shows a clear order among the possible diagnostic indicators in the analytical framework (Figure 11). Stool microplastics had the highest significant value, 0.39, which means they make up 39% of the overall contribution. This means that stool is the most important biomarker in the model. It also means that a single stool measurement may explain around 39% of the difference between high-risk and low-risk countries. Heavy metal load is the second most important part of the biomarker profile, with a significance value of 0.32, or 32%. Chronic inflammation is in third place, with a significance value of 0.19, or 19%. Figure 11 shows that these three indications make up the biggest part of the biomarker contribution pattern. Prostate tissue and blood biomarkers, on the other hand, only add a little bit, with significance values of 0.06 and 0.04, respectively. So, the way the values are spread out suggests a very concentrated classification structure, with microplastic particles in feces, heavy metals that are pollutants, and chronic inflammation being the most likely potential biomarkers in the current analytical framework.

To quantify the uncertainty of these importance values, we refitted the random forest 500 times with different random seeds and recomputed the permutation feature importance on each fit by using the same set of biomarkers. We then summarized the normalized importance of each biomarker as mean ± standard deviation across runs (Table 4). The stool microplastics had by far the highest mean importance (66.7% ± 38.8%), followed by the heavy metal load (22.1% ± 20.2%), whereas the blood microplastics (5.7% ± 8.5%), the prostate tissue microplastics (4.2% ± 7.3%), and the chronic inflammation (1.4% ± 2.8%) contributed substantially less to the model. These large standard deviations confirm that the exact percentages are numerically unstable in such a small ecological dataset, but the ranking of the biomarkers (e.g., stool > heavy metals > other markers) was stable across all runs; accordingly, we interpret the importance values as qualitative indicators of relative influence rather than precise effect estimates.

**Table 4:** Stability of biomarker importance estimates in the random forest model.

Biomarker	Mean_importance_pct	SD_importance_pct
Stool_samples	66.7	38.8
Heavy_metal_load	22.1	20.2
Blood_samples	5.7	8.5
Prostate_tissue	4.2	7.3
Chronic_inflammation	1.4	2.8

### 3.7 Integrated visualization of the analytical results

**Figure 12:**
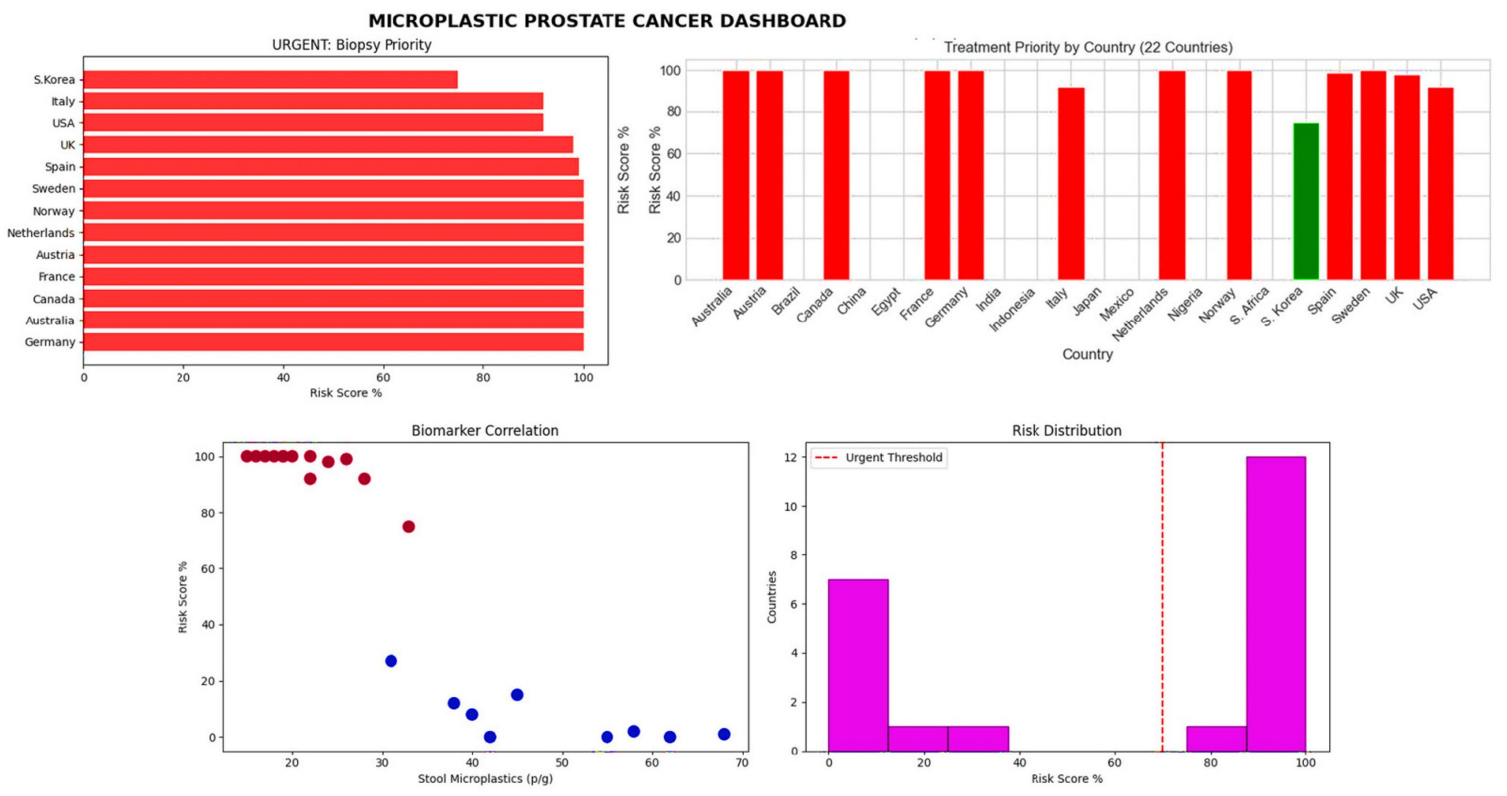
Microplastic prostate cancer dashboard. The dashboard displays multiple aspects of prostate cancer risk, including biopsy and treatment priorities across countries, biomarker correlations with stool microplastic levels, and the distribution of countries based on their risk scores.

The integrated dashboard shows prostate cancer risk trends in a single view across four panels (Figure 12). The top left panel shows the urgent case category (biopsy priority), which makes it easy to see which countries are at the highest risk. This panel depicts a group of 13 countries that are considered urgent. Eight of them have a risk level of 100%, and the other five have scores between 75 and 99%. This distribution is in line with the high-risk group shown before and with the patterns illustrated in the confusion matrix (Figure 8) and the risk matrix (Figure 9). The top right panel shows which countries have the most urgent need for treatment. The red bars on this panel demonstrate that 13 countries are in the “urgent case” category, while the green bars show that the other nine countries are in the “control” category. It is easy to see the difference between these two groups, and it follows the same clustering pattern as the previous findings.

The bottom left panel demonstrates how the levels of microplastics in feces are related to risk scores. There are two separate groups on this panel. Red dots show high-risk countries, which are at the highest risk levels, while blue dots show low-risk countries, which are at the lowest risk levels. This division is in line with the classification structure seen in earlier studies. The lower right panel shows how danger levels are spread out throughout countries. The graph clearly depicts two different groups, with a dashed red vertical line showing the urgency threshold. Countries are split into two groups on either side of this line, which are the urgency and monitoring groups. The fact that these two groupings do not overlap is in line with what was found in past classifications. The four panels in Figure 12 work together to show a clear and cohesive picture of the risk structure at the country level. All of the viewable parts show the same clustering tendencies.

## 4 Discussion

This study examined country-level associations between microplastic exposure indicators and prostate cancer burden across 22 countries. The main finding was that the largest external exposure gradient did not consistently correspond to the gradient of reported prostate cancer incidence. This pattern should be interpreted cautiously. It does not indicate that microplastic exposure is unrelated to prostate cancer (29). It also does not prove that internal retention is the main explanation. A more balanced interpretation is that external exposure indicators alone may be insufficient to explain international variation in reported prostate cancer incidence (44). The observed mismatch may reflect differences in diagnostic capacity, PSA screening intensity, cancer registry completeness, population age structure, lifestyle patterns, genetic susceptibility, and co-exposure to other environmental pollutants.

The results suggest that biological-response indicators may add value when interpreting microplastic-related health risks. Stool microplastic concentrations, heavy metal burden, and chronic inflammation appeared as important variables in the analytical framework (45). These findings are consistent with toxicological evidence linking microplastics and associated pollutants to oxidative stress, inflammatory signaling, DNA damage, endocrine disruption, and altered cellular behavior (46, 47). However, these associations remain exploratory. Stool microplastic levels may reflect recent exposure or excretion rather than retained internal burden (48). Heavy metals may represent co-exposure rather than a microplastic-specific effect (49). Chronic inflammation may also be influenced by infection, metabolic disease, diet, obesity, smoking, and other country-level health determinants (50). Therefore, these markers should be considered candidate indicators for future validation, not confirmed biomarkers of prostate cancer risk.

These findings should also be interpreted in relation to previous population-level research on microplastics and cancer. Existing ecological studies remain limited. Most available research has focused on environmental distribution, human exposure, experimental toxicity, or tissue detection rather than cancer outcomes across populations (51, 52). Compared with earlier work that examined single exposure sources or general cancer-related mechanisms, the present study integrates multiple exposure pathways with biomonitoring and biological-response indicators in one comparative framework (53). This design may help generate new hypotheses about combined exposure profiles and cancer burden. However, it does not overcome the limitations of ecological inference (54, 55). Like other population-level analyses, the present study remains vulnerable to exposure misclassification, ecological fallacy, and residual confounding.

The interpretation of tissue-related evidence also requires caution. Previous studies reporting microplastics in prostate tissue are biologically relevant because they show that particles may be detected in urological tissues (56). However, these studies are usually based on small and non-representative samples. They do not provide nationally representative prostate tissue burden data (57). The present analysis also did not include direct prostate tissue measurements across countries. Therefore, internal retention and tissue-response pathways should be treated as plausible hypotheses. They cannot be used as confirmed explanations for the country-level patterns observed here (58). Future studies should use standardized tissue sampling, polymer identification, particle-size characterization, and individual-level clinical data to test whether tissue microplastic burden is associated with prostate cancer incidence, stage, or progression.

From a policy perspective, the findings support the need to connect environmental monitoring with cancer surveillance. Microplastic exposure may occur through air, drinking water, seafood, and consumer products (59). This makes exposure reduction relevant to several Sustainable Development Goals (SDGs), particularly SDG 3 on good health and well-being, SDG 6 on clean water and sanitation, SDG 12 on responsible consumption and production, and SDG 14 on life below water (60). Improved monitoring of microplastics in drinking-water systems can support safer water management under SDG 6 (61). Better assessment of seafood contamination can strengthen food-safety and consumer-protection actions linked to SDG 12 and SDG 14. Monitoring airborne particles may also support pollution-control strategies that protect population health under SDG 3 (62). In parallel, countries with high reported prostate cancer burden may benefit from stronger integration between environmental exposure assessment, biomonitoring, and cancer registry systems (63). This approach links disease prevention with pollution control, safer consumption, and environmental protection (64). However, these findings should guide surveillance priorities rather than direct policy decisions. Stronger individual-level and longitudinal evidence is still needed before specific interventions can be recommended (65).

This study has several limitations that should guide future research. Its ecological design prevents interpretation at the individual-risk level, and the small sample of 22 countries limits statistical power, model stability, and generalizability. The analysis also did not adjust for major prostate cancer determinants, including age structure, PSA screening intensity, diagnostic access, diet, obesity, smoking, ethnicity, genetic susceptibility, socioeconomic status, and healthcare quality (66). These factors may strongly affect reported prostate cancer incidence. In addition, high classification performance in a small, structured dataset may reflect overfitting, data leakage, or dataset-driven separation rather than true predictive validity (67). Future studies should use larger, individual-level, and longitudinal datasets with standardized microplastic measurements in stool, blood, urine, semen, and prostate tissue (68). They should also assess polymer type, particle size, additives, adsorbed pollutants, tumor stage, inflammatory markers, and molecular features. Such studies are needed to determine whether microplastic exposure contributes to prostate cancer risk or whether the observed country-level patterns mainly reflect detection bias, confounding, or co-exposure effects (69).

## 5 Conclusion

This research created a combined statistical and machine learning framework to evaluate the worldwide correlation between microplastic exposure and prostate cancer burden in 22 nations. The analysis integrated many exposure dimensions, including air inhalation, drinking water consumption, seafood intake, personal care product usage, and stool microplastics, alongside biomonitoring indications, heavy metal load, and cancer burden metrics. The research indicated that the risk of prostate cancer at the country level does not adhere to a straightforward linear exposure–response relationship. Countries exhibiting elevated environmental exposure did not consistently correlate with the highest cancer incidence rates. The analytical findings repeatedly suggested that the internal management of microplastics, especially tissue retention and associated biological burden, may be more significant than external load alone in influencing risk patterns. The model also found that stool microplastics, heavy metal burden, and chronic inflammation were the most useful candidate biomarkers in the framework. The risk matrix and triage structure then divided the countries investigated into unambiguous low-risk and high-risk groups. These results offer a more accurate foundation for understanding the international risk of prostate cancer linked to microplastics. They also show how important it is to use both exposure indicators and biomarker-based data when looking at cancer in the environment. From a practical standpoint, the study endorses the implementation of integrated exposure–biomarker frameworks in environmental monitoring, preliminary risk assessment, and sustainability-focused health planning in analogous places globally. Subsequent research ought to confirm these trends in more extensive and standardized datasets, integrate direct human biomonitoring and tissue-based assessments, and evaluate if retained microplastic load serves as a more reliable predictor of prostate cancer outcomes compared to external exposure metrics alone. Longitudinal research will be particularly crucial for elucidating causal pathways, retention dynamics, and co-exposure effects.

## Data Availability

The original contributions presented in the study are included in the article/supplementary material, further inquiries can be directed to the corresponding author.
